# Standard Reference Materials (SRMs) for the Calibration and Validation of Analytical Methods for PCBs (as Aroclor Mixtures)

**DOI:** 10.6028/jres.109.016

**Published:** 2004-04-01

**Authors:** Dianne L. Poster, Michele M. Schantz, Stefan D. Leigh, Stephen A. Wise

**Affiliations:** National Institute of Standards and Technology, Gaithersburg, MD 20899-0001, USA

**Keywords:** Aroclors, PCBs, Standard Reference Materials (SRMs), transformer oil, water

## Abstract

Six Standard Reference Materials (SRMs®) have been prepared by the National Institute of Standards and Technology (NIST) for the determination of PCBs as different Aroclor mixtures in methanol. Six additional SRMs of the same Aroclors in transformer oil have also been prepared. Specifically, solutions of Aroclors 1016, 1232, 1242, 1254, and 1260 have been gravimetrically prepared (individually) in methanol and transformer oil, mixed, and transferred to amber glass ampoules in approximately 1.2 mL aliquots. Gas chromatography with electron capture detection (GC-ECD) has been used to verify the gravimetric data for each solution and transformer oil SRM. Liquid chromatography was used for the isolation of the Aroclors from the transformer oil SRMs prior to GC-ECD analysis. Separate calibration solutions and oils were prepared with Aroclor levels similar to those in each methanol solution and transformer oil SRM and were processed alongside the samples. The GC-ECD response of each Aroclor was monitored relative to internal standards that were added to the complex mixtures for quantification. The gravimetric concentrations of Aroclors 1242 and 1254 in methanol were also examined by the same method of analysis (GC-ECD) using several different sources of Aroclors and two different capillary GC columns: a 5 % phenyl methylpolysiloxane phase and a relatively non-polar phase. The preparation of the materials, the gas chromatographic results, and the certified concentration values for each Aroclor SRM are described in this paper.

## 1. Introduction and Background

### 1.1 Polychlorinated Biphenyls (PCBs)

PCBs are a class of synthetic, chlorinated organic compounds. Individual congeners are produced by reacting the basic biphenyl structural unit with chlorine, replacing anywhere from 1 to 10 of the original hydrogens with chlorine yielding up to 209 possible arrangements (congeners). Mixtures of the 209 PCB congeners comprised commercial mixtures with the overall mass fraction of chlorine ranging from 20 % to 80 % depending on the manufacturing process. PCBs exhibit wide industrial versatility as a result of their physical properties such as non-flammability, thermal stability, and low reactivity. Various industries have produced PCB-containing commercial products such as dielectric and hydraulic fluids, solvents, and plasticizers. Dielectric fluids were largely used in capacitors and transformers. From 1927 to 1977, commercial mixtures of industrial fluids containing PCBs were solely manufactured in the United States by the Monsanto Chemical Company,^1^ and which also accounted for an estimated 50 % of the worldwide production of PCBs. Monsanto registered its PCB mixtures under the trade name Aroclor [1–3].

The applications associated with PCB mixtures coupled with their physical properties have resulted in the widespread distribution of PCBs within and among environmental compartments. These compounds tend to bioaccumulate [4] and biomagnify [5] in food webs and their toxicology is a critical environmental [6, 7] and human health issue [8–10]. Historical references for the latter topics include observations by Jensen in 1966 [11] and Clayton et al. [12]. The monitoring of PCBs in the environment has been a strong research focus, particularly for drinking water [2, 13–19]. The analysis of drinking water for the determination of PCBs is increasing in Europe [20] and Asia [21]. The dechlorination (i.e., remediation) of PCBs in water [22] and sediment [23] has received attention as well. In addition, PCBs are routinely measured in oil [24, 25], these measurements are often conducted to determine proper disposal mechanisms.

### 1.2 Measurement Standards for Drinking and Wastewater Quality

Since PCBs are ubiquitous in the environment, laboratories that test the quality of water play a key role in ensuring the safety of U.S. water systems. The analysis of drinking and waste water is performed by a large system of laboratories that provide chemical measurement services. The assurance that these services provide accurate results is extremely important. Standard Reference Materials (SRMs^®^) [26] assist with this process. These are materials that have been well-characterized for specific chemical properties such as concentration (denoted as mass fraction) for specific chemical species. Many solution SRMs currently available from NIST are related to regulated chemicals such as PCBs in water although these are prepared in organic solvents. These include the following SRMs: SRM 1493 (PCBs in 2,2,4-Trimethylpentane) [27], SRM 2262 (Chlorinated Biphenyl Congeners in 2,2,4-Trimethylpentane, Nominal Concentration 2 μg mL^−1^) [28] and SRM 2275 (PCB Congener Solution-II in Isooctane) [29]. These solutions are useful for validating chromatographic separations, retention times, and analyte detector response [30]. A wide range of new SRM solutions in more polar solvents, such as acetone, methanol, and water, for regulated chemicals in water that are not presently characterized in existing SRMs have recently been prepared at NIST. For the organic components, these include six individual Aroclors in a water-soluble solvent (methanol) and the same six individual Aroclors in transformer oil (Table 1). Other SRMs for organics in water-soluble solvents include solutions of pesticides, herbicides, phthalates, and organic disinfecting by-products (Table 2). All of these have been gravimetrically prepared and ampouled using an established standard operating procedure. The primary standards used for solution preparation are well characterized, with purity determinations by multiple methods, where possible, except for analytes that comprise mixtures (i.e., Aroclors). The certified mass fraction for each solution SRM is based on gravimetric preparation of the solution, analytical verification of the gravimetry, and purity of the starting material (when applicable). Currently, 26 individual semi-volatile organic SRM solutions and two sets of Aroclor-related SRMs have been prepared (Table 2). Fifteen solutions of volatile organic compounds (VOCs) have also been prepared. The Aroclor-related SRMs (Table 1) are described in this paper.

The new water-soluble solution SRMs are to be used by laboratories that provide proficiency testing to environmental laboratories that monitor regulated chemicals in water [31, 32]. Proficiency testing (previously referred to as performance evaluation), mandated and conducted in the past by the U.S. Environmental Protection Agency (EPA) to support the implementation of national water programs (see next paragraph), indicates a laboratory’s competency to analyze water samples [33]. Results are used to assess a laboratory’s ability to conduct analyses, produce reliable environmental measurements, and serve as a component of the overall federal goal to assure quality in measurements necessary to implement the Clean Water Act and the Safe Drinking Water Act [33]. In addition, results have been used by the U.S. EPA to assess the capability of the nation’s laboratory community to conduct analyses for certain analytes. For example, if results from proficiency testing indicate that a disproportionate number of laboratories did not properly analyze samples for a given analyte, then additional method development was warranted for the nation’s laboratories by the Agency [33]. The U.S. EPA recently transferred portions of its role within the laboratory proficiency testing program to private sector “proficiency testing” providers. Providers are expected to develop and manufacture proficiency testing materials that are in accordance with the SRMs listed in Tables 1 and 2.

There currently are three national water programs that make use of proficiency testing [33]: the Water Supply (WS), the Water Pollution (WP), and the Discharge Monitoring Report Quality Assurance (DMRQA) study programs. The WS program supports the chemical, microbiological, and radiochemical aspects of the Safe Drinking Water Act. The WP program includes chemical proficiency testing which provides testing to laboratories that analyze common surface water quality pollutants and supports State wastewater and other environmental laboratory certification programs. Many States conduct laboratory evaluation (i.e., accreditation) programs in support of the National Pollutant Discharge Elimination System (NPDES) under the Clean Water Act and often require laboratories to participate in the WP program. The DMRQA program includes inorganic chemical components and whole effluent toxicity proficiency testing. It is a tool for assessing the quality of monitoring data submitted by the NPDES. Applicable analytes within each program include a wide range of trace metals and inorganic compounds, asbestos, volatile organic compounds, pesticides, herbicides, polycyclic aromatic hydrocarbons (PAHs), PCBs, dioxins, adipate and phthalate esters, haloacetic acids, chloral hydrate, total organic carbons, alkalinity, calcium hardness, total filterability residue, pH, turbidity, minerals, nutrients, residual chlorine, cyanide, volatile halocarbons, oil and grease, and specific conductance. A complete list of analytes is available [34]. The Aroclor-related SRMs (Table 1) described in this paper comprise components within the WS and WP programs.

### 1.3 Aroclors in Methanol and Transformer Oil

Aroclors 1016, 1232, 1242, 1254, and 1260 have been gravimetrically prepared in methanol and transformer oil, mixed, and transferred to amber glass ampoules in approximately 1.2 mL aliquots. The solutions and oils are available as SRMs (Table 1). A unit of each material consists of five ampoules. Methanol solutions and transformer oils are intended for use in the determination of PCBs in water and oil, respectively. The SRMs are primarily to be used as instrument response calibration solutions and for validating methods of analysis. Researchers involved with environmental monitoring, waste management, and similar activities will likely find the materials useful. In addition, as described in Sec. 1.2, the SRMs are to be used by laboratories that provide proficiency testing to environmental laboratories that measure Aroclors in water and oil. The target levels of Aroclors in each methanol solution and transformer oil were determined in conjunction with U.S. EPA and the WS and WP programs (see Sec. 1.2) based on historical measurements of Aroclors in water and transformer oil, and were designed to allow for an appropriate dilution scheme for calibration and quantification of PCBs in water, oil, or similar matrices. Prior to the development of these Aroclor-related SRMs, SRM 1581 (PCBs in Oil) was available for the determination of the concentrations of PCBs in oil [35]. SRM 1581 consisted of ampoules of Aroclors 1242 and 1260 in both motor and transformer oil at concentrations near 100 μg g^−1^,. The material was issued in 1982 and the Certificate of Analysis was revised in 1990. The supply of this material is now depleted. SRM 1581 will be replaced by six individual Aroclors in Transformer Oil SRMs (SRMs 3075 through 3080, Table 1). The preparation and analysis of the new methanol and transformer oil SRMs are described in this paper along with the resulting certified Aroclor mass and volume fraction data for each material.

## 2. Materials and Methods

### 2.1 Preparation of Solution and Oil SRMs

With the exception of Aroclor 1016, which was obtained from a commercial source (Supelco, Supelco Park, Bellefonte, PA), the Aroclors were obtained from the former U.S. EPA repository (Research Triangle Park, NC). All solutions and transformer oil SRMs were prepared at NIST. Prior to each solution and oil preparation, glassware was washed, dried, and baked at 500 °C for 18 h. In addition, about 45 gross soft glass ampoules were cleaned with distilled water and air dried.

The methanol solutions and transformer oils were prepared by weighing and mixing the Aroclors and the methanol or transformer oil in a glass bottle (10 L). The bottle was sealed with an inert stopper and was covered with dark plastic to shield the solution from light. An electronic microbalance was used to determine the mass of each Aroclor added. Specifically, a weighed aliquot of each Aroclor, contained within an aluminum weigh boat or a glass volumetric cylinder, was transferred to the bottle to which approximately 100 mL of methanol or transformer oil had been added. After the Aroclor was added, methanol (chromatographic grade) or transformer oil (Univolt 60, Exxon) was added to the bottle (approximately 9 L). The total mass of the solution was determined using a top-loading balance and was corrected for the mass of the aluminum weigh boats when necessary. Each solution and transformer oil was stirred overnight. Prior to starting the preparation of each solution or transformer oil, the balances were calibrated and zeroed.

Individual units of each solution or transformer oil were ampouled at NIST. Immediately prior to ampouling, the combined mass of the solution or transformer oil, bottle, and stir bar was recorded. This mass is used for the calculation of the gravimetric mass fractions for each analyte (Table 1). Ampoules were filled with argon prior to filling them with the solution. Immediately following the filling, each ampoule was flame sealed. Each ampoule contains about 0.95 g (approximately 1.2 mL) of methanol solution or about 1.2 g (approximately 1.4 mL) of transformer oil.

### 2.2 Analysis of Solutions and Transformer Oils

**Fig. 1:**
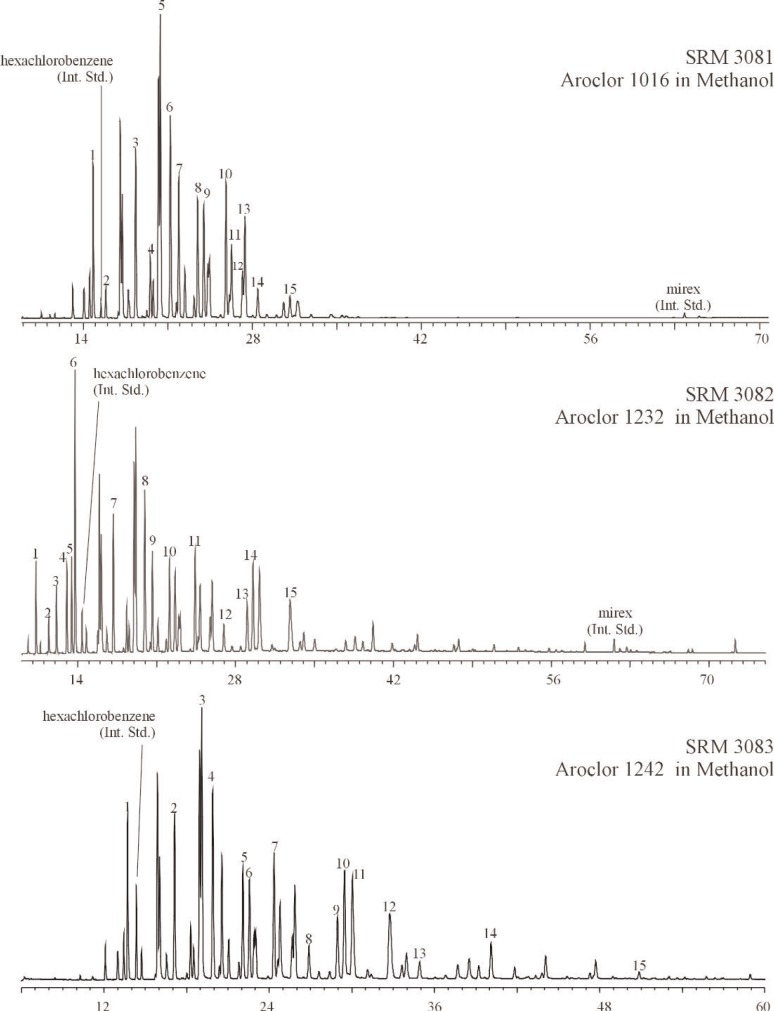

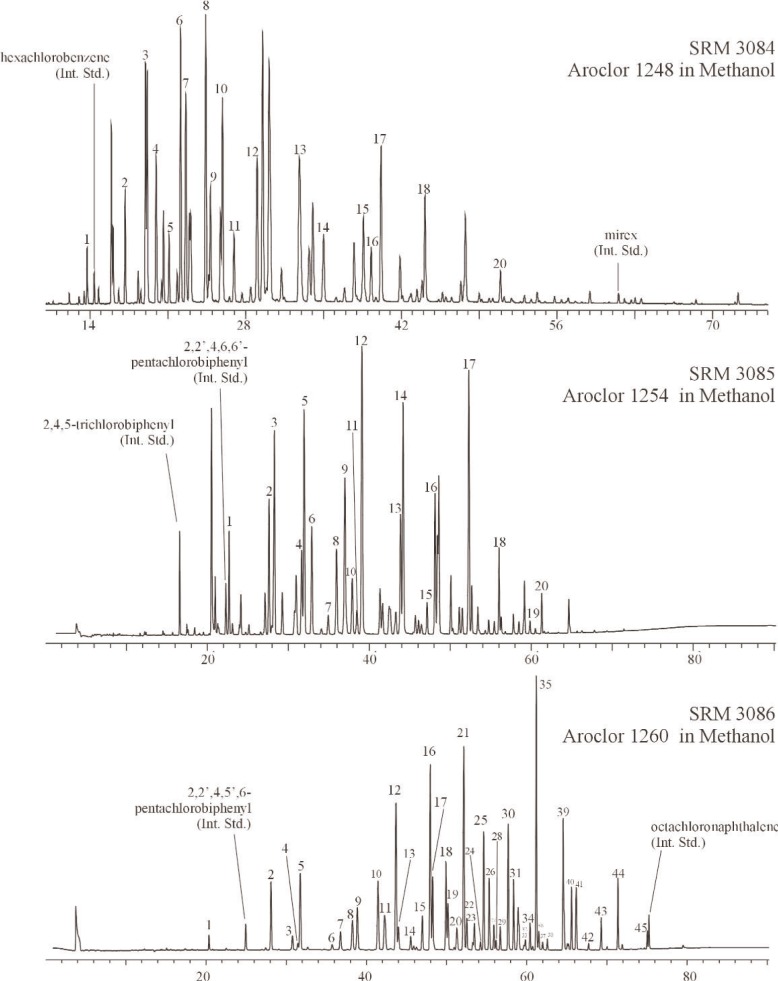
Gas chromatograms from the analysis of Aroclors in methanol solutions using a 5 % phenyl methylpolysiloxane phase (DB-5 60 m × 0.25 mm; 0.25 μm film thickness). Temperature programs are in Table 1. The peaks used for quantification of the Aroclor mass in each solution are shown. (Int. Std.) = internal standard, *x*-axis represents time in minutes.

**Fig. 2:**
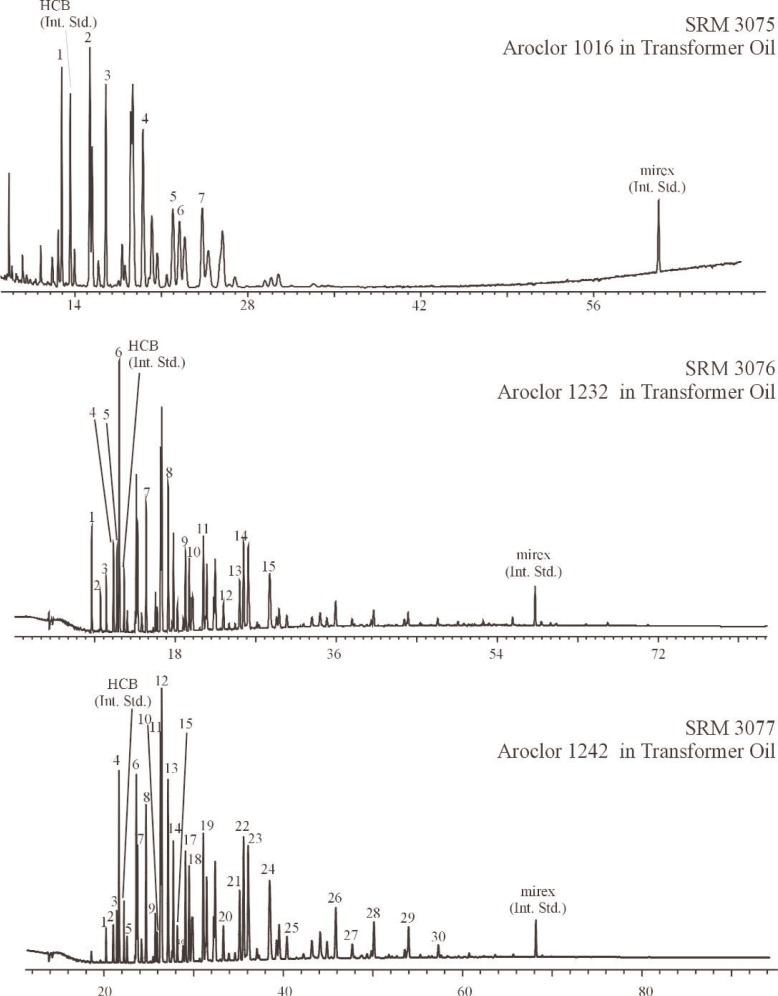

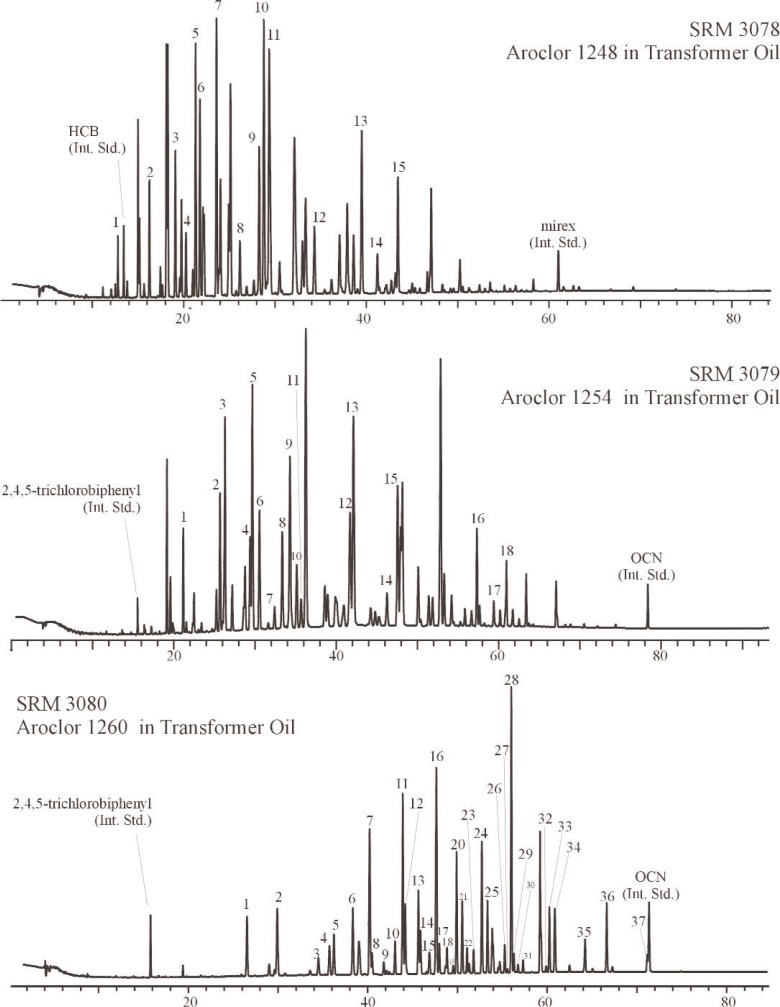
Gas chromatograms from the analysis of Aroclors in transformer oils using a 5 % phenyl methylpolysiloxane phase (DB-5 60 m × 0.25 mm; 0.25 μm film thickness). Temperature programs are in Table 1. The peaks used for quantification of the Aroclor mass in each solution are shown. (Int. Std.) = internal standard, *x*-axis represents time in minutes.

The gravimetrically determined concentrations in each solution and transformer oil were verified using gas chromatography with electron capture detection (GC-ECD). Prior to GC-ECD analysis the Aroclors were isolated from the transformer oil SRMs using liquid chromatography (see Sec. 2.2.2). For both the solutions and transformer oils, nine ampoules were selected from the entire lot of ampoules for each SRM (Table 1) using a stratified, random sampling scheme. Four calibration solutions (methanol) and four calibration transformer oils were prepared gravimetrically at concentrations (Table 1) near those of the original ampouled solution or transformer oil for each SRM. In addition, a gravimetric solution of two compounds not detected in each Aroclor was prepared for each analysis for use as an internal standard solution (methanol) or transformer oil (Figs. 1 and 2). Upon the opening of each SRM ampoule, a single aliquot of solution or transformer oil was gravimetrically transferred to an amber autosampler vial and capped. In addition, an aliquot of the internal standard solution or transformer oil was gravimetrically added to each autosampler vial for quantitation purposes.

#### 2.2.1 Methanol Solutions

All methanol solutions were analyzed directly by GC-ECD using a 5 % (mole fraction) phenyl methylpolysiloxane capillary column (DB-5, J&W Scientific, Folsom, CA; 60 m × 0.25 mm, 0.25 μm film thickness). Additional analyses (see Sec. 2.2.3) were conducted on selected SRMs using a second capillary column (a relatively non-polar phase, DB-XLB, J&W Scientific, Folsom, CA; 60 m × 0.25 mm, 0.25 μm film thickness). The four calibration solutions (in methanol) prepared for each SRM were chromatographed in concert with the samples that corresponded to each SRM to measure a response factor for each Aroclor relative to the internal standards. Samples (*n* = 9) were analyzed in duplicate. GC-ECD temperature programs are given in Table 1 for each SRM, these varied in accordance with optimum PCB congener separations for each Aroclor. GC-ECD chromatograms of each Aroclor in Methanol SRM on the 5 % phenyl methylpolysiloxane capillary column are given in Fig. 1.

#### 2.2.2 Transformer Oils

Aroclors were isolated from the transformer oil SRM aliquots and calibration oils using liquid chromatography prior to GC analysis. Specifically, transformer oil SRM aliquots (prepared with internal standards as described for the methanol solutions) were transferred to an aminopropyl solid phase extraction (SPE) column for an initial isolation of the Aroclors from the transformer oil using hexane as the mobile phase. Eluants were concentrated (via nitrogen evaporation) and the Aroclors were further isolated from the oil matrix using a semi-preparative aminopropylsilane column using hexane as the mobile phase [36, 37]. Eluants were concentrated and analyzed by GC-ECD for the determination of the concentration of Aroclor in each transformer oil SRM. A 5 % phenyl methylpolysiloxane capillary column (described above) was used with samples analyzed in triplicate. The four calibration oils prepared for each transformer oil SRM were processed alongside the aliquots of the SRM transformer oils as described above. GC-ECD temperature programs are given in Table 1 for each transformer oil SRM and GC-ECD chromatograms are given in Fig. 2. Two control transformer oils were also analyzed for the determination of the concentration of Aroclors 1242 and 1260 in transformer oil. Specifically, aliquots of Aroclor 1242 and Aroclor 1260 in SRM 1581 (PCBs in Oil) [35] were prepared and analyzed as described above. The analytically determined concentrations (*n* = 3) of Aroclors 1242 and 1260 in SRM 1581 were [99 (1)] μg g^−1^ and [108 (4)] μg g^−1^, respectively, where the value in parentheses is the standard deviation of replicate measurements. Results are similar to the reported values on the SRM 1581 Certificate of Analysis: (100 ± 1) μg g^−1^ (Aroclor 1242) and (100 ± 3) μg g^−1^ (Aroclor 1260).

#### 2.2.3 Additional Analyses

##### 2.2.3.1 Analytical Measurements Using Aroclors From Different Sources

To determine the effect, if any, of using sources of Aroclors different from those used to prepare the Aroclor-related SRMs, the gravimetric concentrations of Aroclors in selected methanol SRMs were determined via GC-ECD using Aroclors obtained from several different sources. Specifically, for SRM 3083 (Aroclor 1242 in Methanol), nine ampoules of SRM 3083 were used for the determination of the concentration of Aroclor 1242 in SRM 3083 alongside four individual calibration solutions (in methanol) prepared from each of four sources (AccuStandard, New Haven, CT; Ultra Scientific, North Kingstown, RI; U.S. Food and Drug Administration (FDA); U.S. EPA). The U.S. FDA Aroclor 1242 was previously used to prepare SRM 1581, PCBs in Oil [35]. The U.S. EPA Aroclor 1242 was used to prepare SRM 3083, Aroclor 1242 in Methanol (Table 1). The calibration solutions were prepared gravimetrically at concentrations near that of the original ampouled solution (16.43 μg g^−1^, Table 1). A gravimetric solution of 4-monochlorobiphenyl and 2,4,6-trichlorobiphenyl was also prepared for use as an internal standard solution. These two compounds were not observed in SRM 3083 by GC-ECD and did not coelute with other PCB congeners present in SRM 3083 on a 5 % phenyl methylpolysiloxane phase or a relatively non-polar phase (described above in Sec. 2.2.1 and below in Sec. 2.2.3.2). The 16 calibration standards (4 for each of 4 sources) were analyzed on the 5 % phenyl methylpolysiloxane phase to measure the Aroclor 1242 response factor relative to each internal standard. Table 3 describes the GC conditions, Fig. 3A provides a GC-ECD chromatogram of SRM 3083, and Fig. 4A provides chromatograms of Aroclor 1242 from each of the four sources.

**Fig. 5:**
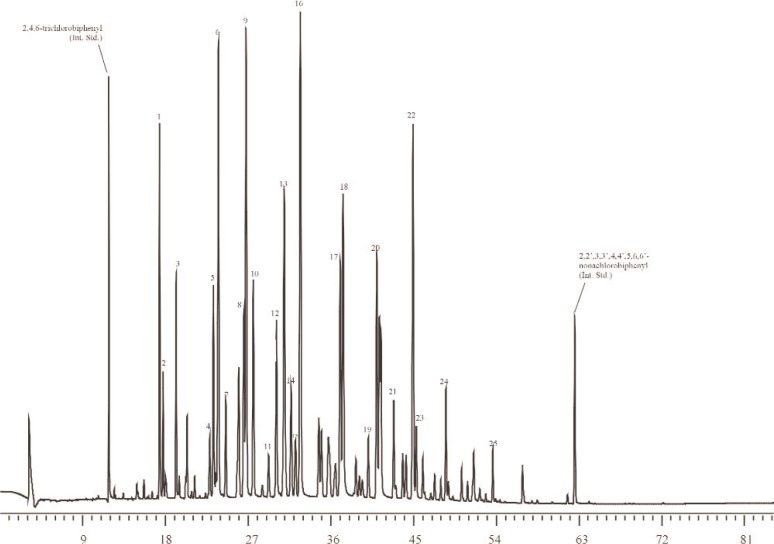
Gas chromatogram from the analysis of Aroclor 1254 in SRM 3085 using a 5 % phenyl methylpolysiloxane phase (DB-5 60 m × 0.25 mm; 0.25 μm film thickness) in conjunction with the use of Aroclor 1254 from different sources (see Fig. 6). The peaks used for quantification of the Aroclor mass in solution are shown, *x*-axis represents time in minutes.

**Fig. 6:**
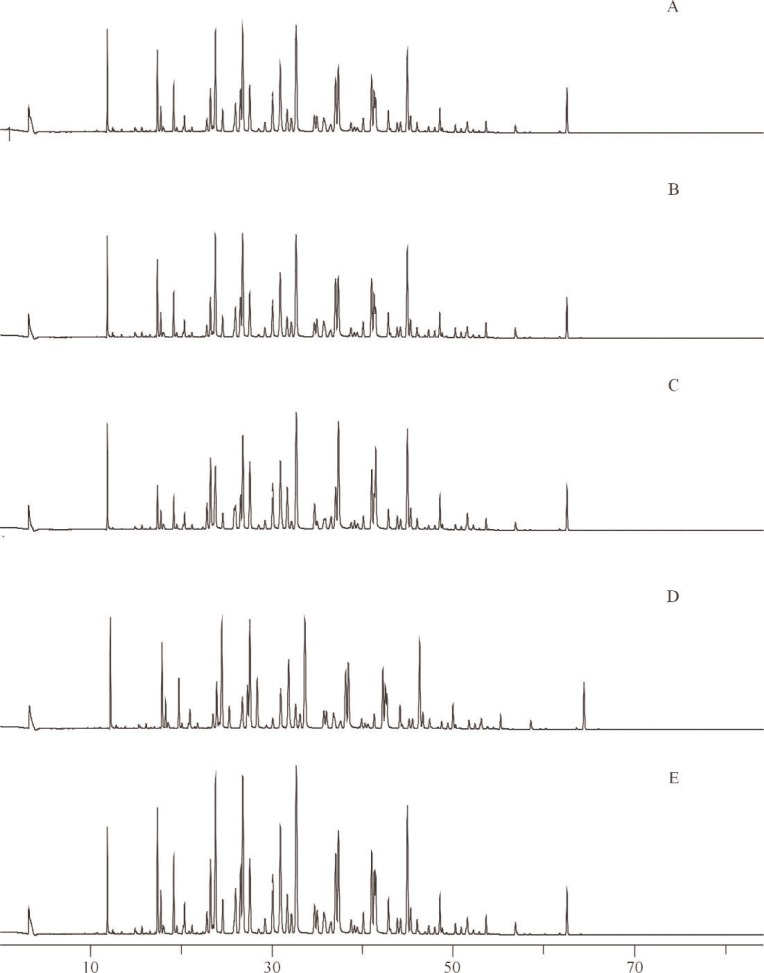
Gas chromatograms of Aroclor 1254 from five different sources on a 5 % phenyl methylpolysiloxane phase (DB-5 60 m × 0.25 mm; 0.25 μm film thickness), *x*-axis represents time in minutes. A: AccuStandard (New Haven, CT), B: Ultra Scientific (North Kingstown, RI), C: Alltech (Deerfield, IL), D: U.S. FDA, E: U.S.

The concentration of Aroclor 1254 in SRM 3085 was also determined using different sources of Aroclors for calibration solutions. Specifically, five ampoules of SRM 3085 were used for the determination of the concentration of Aroclor 1254 in SRM 3085 using different sources of Aroclors. Three calibration solutions for each of five sources (AccuStandard, Ultra Scientific, Alltech (Deerfield, IL), U.S. FDA, U.S. EPA) were prepared gravimetrically at concentrations near that of the original ampouled solution (7.07 μg g^−1^, Table 1). The U.S. FDA Aroclor 1254 was previously used to prepare SRM 1581, PCBs in Oil [35]. The U.S. EPA Aroclor 1254 was used to prepare SRM 3085, Aroclor 1254 in Methanol (Table 1). A gravimetric solution of 2,4,6-trichlorobiphenyl and 2,2′,3,3′,4,4′,5,6,6′-nonachlorobiphenyl was also prepared for use as an internal standard solution. These compounds were not observed in SRM 3085 by GC-ECD and did not coelute with other PCB congeners present in SRM 3085 on a 5 % phenyl methylpolysiloxane capillary GC column. The 15 calibration standards (3 for each of 5 sources) were chromatographed to measure the Aroclor 1254 response factor relative to each internal standard. Table 3 describes the GC conditions and Fig. 5 provides a GC-ECD chromatogram of SRM 3085. In addition, Fig. 6 provides chromatograms of Aroclor 1254 from each of the five sources chromatographed on a 5 % phenyl methylpolysiloxane capillary GC column.

##### 2.2.3.2 Analytical Measurements Using an Additional GC Column

The 9 samples and 16 calibration standards (4 for each of 4 sources) of SRM 3083, Aroclor 1242 in Methanol, prepared as described above (Sec. 2.2.3.1) were also examined using an additional GC column. Specifically, the concentration of Aroclor 1242 in SRM 3083 was determined using a relatively non-polar phase (described in Sec. 2.2.1). Table 3 describes the GC conditions and Fig. 3B provides a GC-ECD chromatogram of SRM 3083 on this column. In addition, Fig. 4B provides chromatograms of Aroclor 1242 from each of the four sources described in Sec. 2.2.3.1 obtained using this second column.

##### 2.2.3.3 Density Measurements

Six ampoules of each methanol solution and transformer oil SRM were used for the determination of the densities of each Aroclor in each SRM. Upon the opening of each ampoule, 1.0 mL of solution was pulled into a gastight syringe and weighed. The mass was recorded and the solution or oil was expelled from the syringe. The syringe was then weighed and the mass was recorded. The density was calculated as the difference between the mass of the syringe full and empty. Different, clean syringes were used for each SRM, as well as different, clean syringes within each set of six measurements for each methanol solution or transformer oil.

## 3. Results and Discussion

### 3.1 Analytical Measurements

The analytical determination of the concentrations of the Aroclors in SRMs 3075 through 3086 is presented in Table 1. These values are based on the areas of the dominant Aroclor PCB peaks (observed via GC-ECD) and the internal standard peaks (Figs. 1–3, 5). This approach is similar to U.S. EPA Method 505 (Analysis of organohalide pesticides and commercial polychlorinated biphenyl (PCB) products in water by microextraction and gas chromatography, revision 2.0) [19]. Method 505 is typically used for the determination of PCBs in water by laboratories that perform chemical analyses of water for U.S. EPA. This approach was originally presented by Web and McCall [38]. A common application of this approach is when PCBs in environmental samples are identified by comparing the PCB congener distribution pattern present in the samples with those obtained from commercial Aroclors. For example, a PCB pattern-matching approach was used to confirm that the source of PCBs present in contaminated feed was transformer oil [39]. Total Aroclor quantitative approaches have been reported for the determination of PCB levels in seafood, serum, sediment, and water, where the PCB content is ultimately expressed in terms of the matched Aroclor mixture [40–44]. Homolog patterns observed in environmental samples are also often compared to those in Aroclor mixtures to assess the type of Aroclor present [45]. A full description of Method 505 is available [19].

Concentrations of each Aroclor are calculated relative to each internal standard and then averaged for each sample and injection. Table 1 lists the analytically determined concentration of each Aroclor in the methanol and transformer oil SRMs. These are compared to the calculated gravimetric concentrations determined during the preparation of each SRM in Table 1. The analytically determined concentrations in general display good agreement with the calculated gravimetric concentrations. The percent difference between the calculated gravimetric concentrations and the analytically determined concentrations ranges from less than 1 % to 13 % with only two above 5 %. The average percent difference between the calculated gravimetric concentrations and the analytically determined concentrations of Aroclors in both the methanol solutions and transformer oils is 3 % and 5 %, respectively.

### 3.2 Additional Measurements

#### 3.2.1 Data from Different Sources of Aroclors

Technical mixtures such as Aroclor were generally manufactured in many different batches. In some instances there may have been sufficient variation in the manufacturing process to produce a substantially different product. To investigate the effect, if any, on the use of Aroclor standards from different commercial sources (that may have Aroclors from different batches) the concentrations of Aroclor 1242 in SRM 3083 and Aroclor 1254 in SRM 3085 were determined using the Aroclors from four and five different commercial sources, respectively (see Sec. 2.2.3.1 and Table 3). Figures 4 and 6 provide chromatograms of Aroclors 1242 and 1254 from each of the sources of Aroclors. For both SRM 3083 and SRM 3085 the mean values from the different sources are similar. The relative standard deviations of the concentrations of each Aroclor across multiple sources is less than 2 % (Table 3). More importantly, the mean concentrations across sources are similar to the SRM gravimetric values. For example, the gravimetric concentration value for Aroclor 1242 in SRM 3083 is 16.43 μg g^−1^ (Table 1) and the mean value determined using four different sources of Aroclor 1242 is 16.23 μg g^−1^ with a standard deviation of 0.30 (Table 3). The agreement is even closer for Aroclor 1254. The gravimetric concentration value for Aroclor 1254 in SRM 3085 is 7.07 μg g^−1^ (Table 1) and the mean value determined using different sources of Aroclor 1242 is 7.063 with a standard deviation of 0.064 (Table 3). The uncertainties of the certified mass fraction values (Sec. 3.3) of the Aroclor-related SRMs account for the use of Aroclors from suppliers other than those used to prepare the Aroclor-related SRMs. Compare the mean concentration values determined using Aroclors from different suppliers (Table 3) with the uncertainties of the certified mass fraction values (Sec. 3.3, Table 4). The mean values are within the uncertainty intervals.

#### 3.2.2 Data from Different GC Columns

Capillary GC columns provide excellent separations of PCB congeners with low background interference, which facilitates accurate quantitation. The 5 % phenyl methylpolysiloxane stationary phase efficiently separates PCB congeners. The use of this column as part of the analytical scheme for the certification PCB concentration values in environmental natural-matrix SRMs has been documented [30]: SRM 1941a, Organics in Marine Sediment [46], SRM 1945, Organics in Whale Blubber [47], SRM 1974a, Organics in Mussel Tissue (Mytilus edulis) [48], SRM 1649a, Urban Dust [49], and SRM 1946, Lake Michigan Fish Tissue [50]. In addition, this column was used for the determination of the concentrations of PCBs in water as part of an experimental scheme to measure and predict PCB congener Henry’s law constants [51, 52]. Solute retention on this column results primarily from dispersion interactions between the solute and stationary phase, and the resulting separations are mostly based on boiling point differences. However, when boiling point differences are subtle, some separations may be hindered. The PCB congener pairs 66 and 95 and 138 and 163 coelute on the 5 % phenyl methylpolysiloxane column [50]. The use of columns with different stationary phases often provides different separation selectivity of organic compounds. This is the case for a range of PAHs [53, 54]. The PCB congener pairs mentioned above can be separated on a column other than the 5 % phenyl methylpolysiloxane column. A relatively non-polar stationary phase (DB-XLB, described in Sec. 2.2.1 and 2.2.3.2) provides separation of PCB congener pairs 66 and 95 and 138 and 163. The concentrations can be determined individually for each PCB congener even with an electron capture detector [50].

Due to subtle differences in selectivity such as those described above, the use of two capillary columns with different selectivity was evaluated for the determination of the concentrations of Aroclors in the Aroclor-related SRMs. Specifically, the concentration of Aroclor 1242 in SRM 3083 was determined using the 5 % phenyl methylpolysiloxane and a relatively non-polar column (see Sec. 2.2.3.2). Figures 3 and 4 provide chromatograms of Aroclor 1242 on both columns. The mean values determined from the two columns are similar (the percent difference between the mean values determined using both columns is on average less than 1 %, Table 3). More importantly, the concentrations of Aroclor 1242 in SRM 3083 determined using either the 5 % phenyl methylpolysiloxane column or the relatively non-polar column are similar to the gravimetric data for SRM 3083 (Table 3). As observed with the use of Aroclors from suppliers other than those used to prepare the Aroclor-related SRMs, the mean concentration values determined using different GC columns (Table 3) are within the uncertainty intervals of certified mass fraction values (see next section and Table 4).

### 3.3 Certified Mass and Volume Fraction Values

Results in Table 1 and 3 were combined to generate certified values for the concentrations of Aroclors in Methanol and Transformer Oil SRMs [55, 56] (Table 4). Each gravimetric value with a conservative standard error estimate based on balance linearity and other type B components was combined with the corresponding analytical result and its standard error. The concentration of Aroclor for each SRM is expressed as the value ± the uncertainty. The certified value is taken to be the unweighted average of the concentrations determined by gravimetric and gas chromatographic measurements. The expanded uncertainty, at the 95 % level of confidence, is calculated as *U* = *ku*_c_, where *u*_c_ is a combined standard uncertainty calculated according to the ISO Guide [57–59] and *k* = 2 is the coverage factor. The value of uc explicitly includes an allowance for differences between the concentration determined by gas chromatographic measurements for various sources of Aroclors and gravimetric preparation. The volume fraction form of the concentrations (in mg L^−1^ or g L^−1^) in Table 4 were obtained by multiplying the certified values, expressed as mass fractions, by the measured density of the methanol solution or transformer oil SRMs. These values are (0.800 ± 0.015) g mL^−1^ or (0.891 ± 0.021) g mL^−1^, respectively. The uncertainties of the density values represent one standard deviation (1 σ) and these are incorporated in the volume fraction uncertainties for the methanol solution and transformer oil SRMs via propagation of error.

### 3.4 Summary

Twelve new Aroclor-related SRMs have been prepared and certified for the concentration of Aroclor in transformer oil (SRMs 3075 through 3080) or methanol (SRM 3081 through 3086). SRM 1581, PCBs in Oil, which is no longer available, will be replaced by the new Aroclor in Transformer Oil SRMs. All of these materials have been designed to assist in the accurate determination of the concentration of PCBs in oil or water. The materials are useful as controls when analyzed alongside samples with unknown quantities of Aroclors. SRMs 3075 – 3086 will be beneficial to laboratories as they focus attention on the accurate determination of Aroclors in environmental samples or validate their own methods of analyses for the determination of Aroclor and PCB mixtures.

## Figures and Tables

**Fig. 3A:**
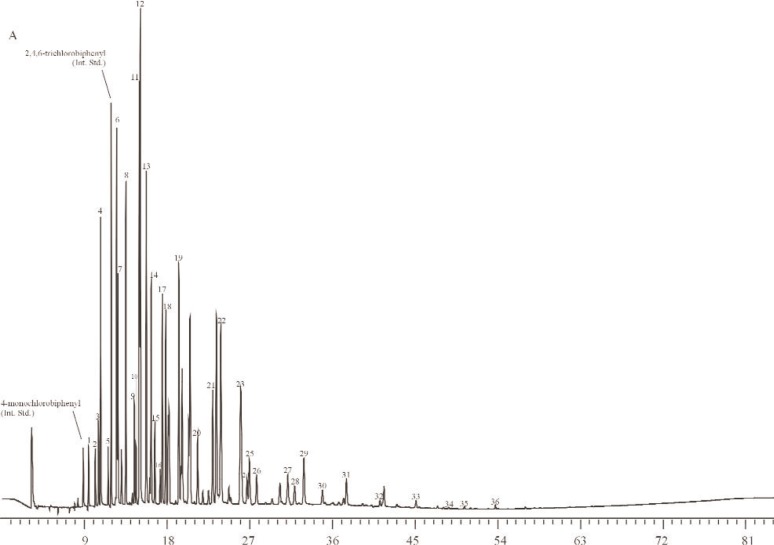
Gas chromatogram from the analysis of Aroclor 1242 in SRM 3083 on a 5 % phenyl methylpolysiloxane phase (DB-5 60 m × 0.25 mm; 0.25 μm film thickness). Temperature program is in Table 3. The peaks used for quantification of the Aroclor mass in solution are shown, *x*-axis represents time in minutes.

**Fig. 3B:**
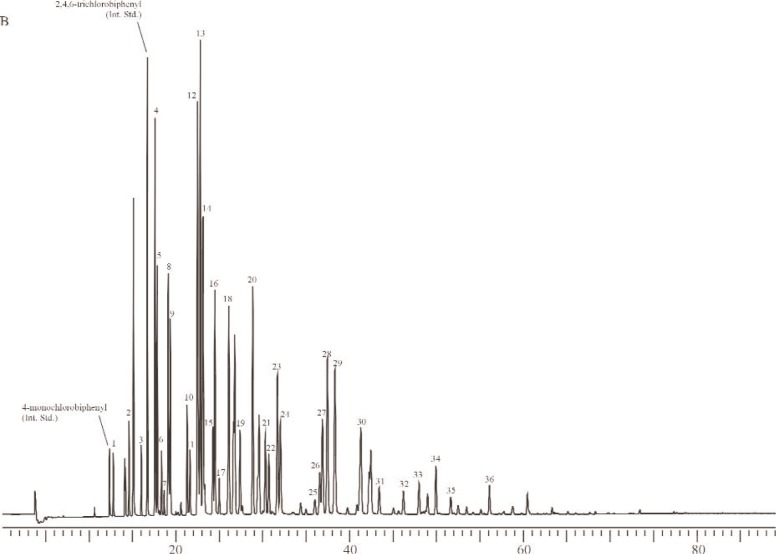
Gas chromatogram from the analysis of Aroclor 1242 in SRM 3083 on relatively non-polar phase (DB-XLB 60 m × 0.25 mm; 0.25 μm film thickness). Temperature program is in Table 3. The peaks used for quantification of the Aroclor mass in each solution are shown, *x*-axis represents time in minutes.

**Fig. 4A:**
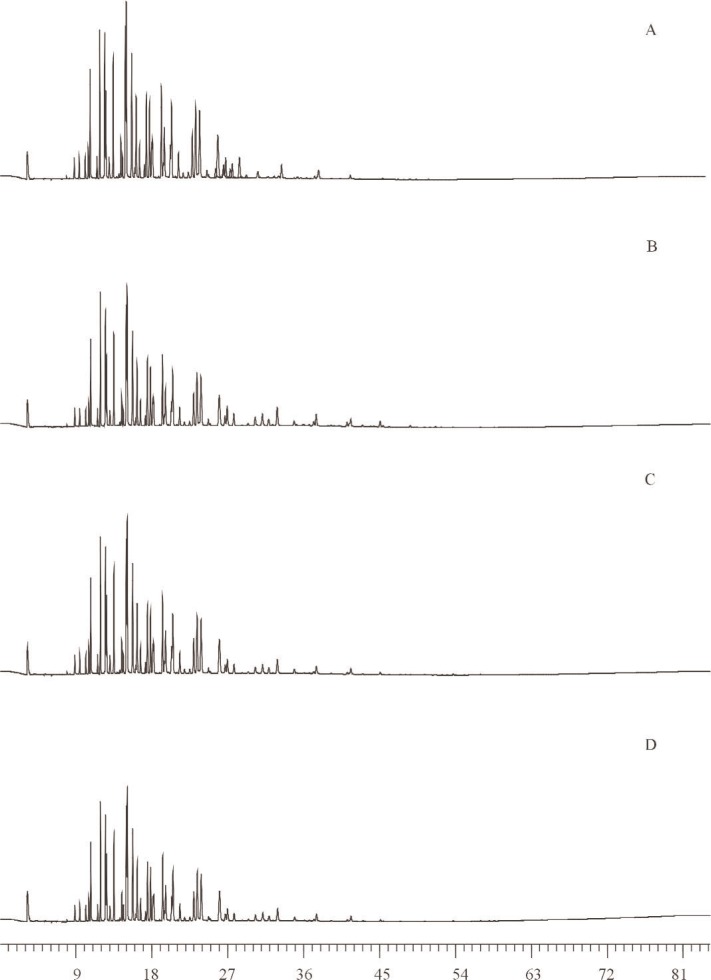
Gas chromatograms of Aroclor 1242 from four different sources on a 5 % phenyl methylpolysiloxane phase (DB-5 60 m × 0.25 mm; 0.25 μm film thickness), *x*-axis represents time in minutes. A: AccuStandard (New Haven, CT), B: Ultra Scientific (North Kingstown, RI), C: U.S. FDA, and D: U.S. EPA.

**Fig. 4B:**
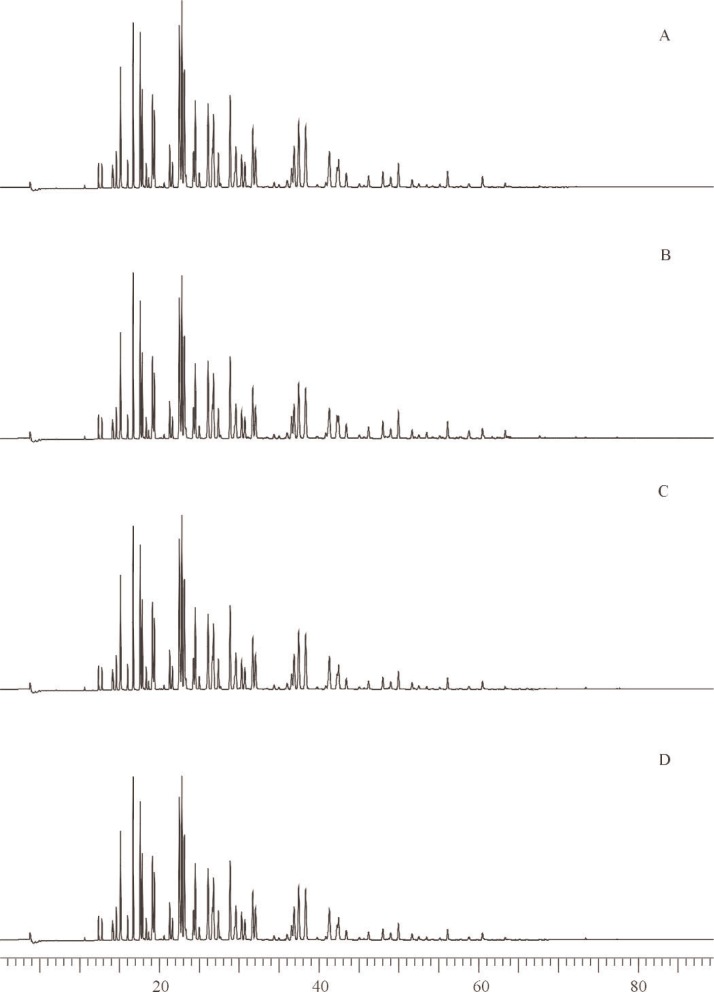
Gas chromatograms of Aroclor 1242 from four different sources on a relatively non-polar phase (DB-XLB 60 m × 0.25 mm; 0.25 μm film thickness), *x*-axis represents time in minutes. A: AccuStandard, B: Ultra Scientific, C: U.S. FDA, and D: U.S. EPA.

**Table 1:** Aroclor-related Standard Reference Materials

SRM No.	SRM Title	Gravimetric Concentration^a^	Analytical concentration^b^
Methanol solutions			
3081	Aroclor 1016 in Methanol	17.19 μg g^−1^	17.01(0.81) μg g^−1,c^
3082	Aroclor 1232 in Methanol	5.04 μg g^−1^	5.46(0.17) μg g^−1,c^
3083	Aroclor 1242 in Methanol	16.43 μg g^−1^	16.57(0.14) μg g^−1,c^
3084	Aroclor 1248 in Methanol	6.97 μg g^−1^	6.815(0.040) μg g^−1,c^
3085	Aroclor 1254 in Methanol	7.07 μg g^−1^	7.22(0.25) μg g^−1,d^
3086	Aroclor 1260 in Methanol	6.22 μg g^−1^	6.139(0.059) μg g^−1,d^
Transformer oils			
3075	Aroclor 1016 in Transformer Oil	16.68 μg g^−1^	17.4(1.6) μg g^−1,e^
3076	Aroclor 1232 in Transformer Oil	4.23 mg g^−1^	4.28(0.17) mg g^−1,e^
3077	Aroclor 1242 in Transformer Oil	4.10 mg g^−1^	4.102(0.084) mg g^−1,e^
3078	Aroclor 1248 in Transformer Oil	3.74 mg g^−1^	3.56(0.10) mg g^−1,e^
3079	Aroclor 1254 in Transformer Oil	3.50 mg g^−1^	3.66(0.11) mg g^−1,f^
3080	Aroclor 1260 in Transformer Oil	1.15 mg g^−1^	1.005(0.024) mg g^−1,e^
Sets of Aroclors^g^			
3091	Aroclors in Methanol	SRMs 3081 – 3086	
3090	Aroclors in Transformer Oil	SRMs 3075 – 3080	

a Concentration calculated based on the mass of the Aroclor added to the mass of the methanol or transformer oil.

b Concentrations determined by GC-ECD and a 5 % phenyl methylpolysiloxane column, the uncertainties listed in parentheses represent one standard deviation of the mean and are based only on the within-method variability.

c GC program: 60 °C (1 min) to 200 °C at 45 °C/min (30 min) to 280 °C at 2 °C/min (15 min).

d GC program: 100 °C (1 min) to 200 °C at 45 °C/min (35 min) to 280 °C at 2 °C/min (12 min).

e GC program: 100 °C (1 min) to 200 °C at 45 °C/min (30 min) to 248 °C to 270 °C at 1°C/min (5 min).

F GC program: 100 °C (1 min) to 200 °C at 45 °C/min (40 min) to 280 °C at 2 °C/min (10 min).

g One vial of each methanol solution or transformer oil comprises SRM 3091 or SRM 3090.

**Table 2:** Newly developed semi-volatile Standard Reference Materials in support of measurements of chemicals in water

SRM	Title	Constituents
3061	Chloral Hydrate in Methanol	chloral hydrate
3062	Haloacetic Acid Mixture in Methanol	bromochloro-; dibromochloro-; dichloro-; monobromo-; monochloro-; trichloro-
3063	Dioxin in Methanol	2,3,7,8-tetrachlorodioxin
3064	Endothall in Water	endothall
3065	Chlorinated Herbicides I in Methanol	acifluorfen; 2,4-D; 2,4-D butyl ester; daiapon; dicamba; picioram; 2,4,5-TP (Silvex); bentazon
3066	Chlorinated Herbicides II in Methanol	dinoseb; pentachlorophenol; 2,4,5-T
3067	Toxaphene in Methanol	toxaphene
3068	Total Chlordane in Methanol	chlordane
3069	Organochlorine Pesticides I in Acetone	aldrin; dieldrin; endrin; heptachlor; heptachlor epoxide; hexachlorobenzene, hexachlorocyclopentadiene; lindane, methoxychlor; propachlor; trifluralin; 4,4′-DDE; 4,4′-DDD; 4,4′-DDT; *ci*s- and *trans*-nonachlor; *cis*- and *trans*-chlordane; endosulfan-I, II, and sulfate; α-, β-, and δ-hexachlorocyclohexane
3070	Organochlorine Pesticides II in Acetone	alachlor; atrazine; simazine; bromacil; butachlor; metolachlor; metribuzin; prometon
3071	Glyphosate in Water	glyphosate
3072	Diquat Dibromide in Water	diquat dibromide
3073	Carbamates and Vydate in Acetonitrile	aldicarb, aldicarb sulfone and sulfoxide; carbofuran; methomyl; oxamy
3074	Adipate and Phthalates in Methanol	di(2-ethylhexyl) adipate and phthalate; dimethyl, diethyl, di-*n*-butyl, butyl benzyl, and di-*n*-octyl phalate
3075–3086	Aroclors in Transformer Oil and Methanol	See Table 1
3090–3091	Set of Aroclors in Transformer Oil and Methanol	See Table 1

**Table 3:** Analytically determined concentrations of Aroclors in methanol determined using different sources of Aroclor Standards and different gas chromatography columns

	Mean μg g^−1^		Mean μg g^−1^	
**Aroclor 1242 (SRM 3083)**				

	5 % phenyl methylpolysiloxane colummn^a^		Relatively non-polar column^b^	
Commercial #1	16.0^c^	(0.3)^c^	15.9^d^	(0.2)^d^
Commercial #2	16.0	(0.3)	16.1	(0.2)
U.S. FDA	16.6	(0.3)	16.2	(0.3)
U.S. EPA	16.4	(0.3)	16.4	(0.2)
mean value (*n* = 4)^e^:	16.2	(0.3)	16.1	(0.2)
gravimetric value^f^:	16.43			

**Aroclor 1254 (SRM 3085)**				

	5 % phenyl methylpolysiloxane column^a^			
Commercial #1	6.96^g^	(0.04)^g^		
Commercial #2	7.09	(0.05)		
Commercial #3	7.14	(0.03)		
U.S. FDA	7.05	(0.07)		
U.S. EPA	7.08	(0.05)		
mean value (n = 5)^e^:	7.06	(0.06)		
gravimetric value^f^:	7.07			

a DB-5 (J&W Scientific, Folsom, CA); 60 m × 0.25 mm, 0.25 μm film thickness, GC program: 100 °C (1 min) to 200 °C at 45 °C/min (30 min) to 248 °C at 2 °C/min to 270 °C at 1 °C/min (5 min).

b DB-XLB (J&W Scientific, Folsom, CA); 60 m × 0.25 mm, 0.25 μm film thickness, GC program: 100 °C (2 min) to 200 °C at 40 °C/min (35 min) to 260 °C at 1.5 °C/min (10 min).

c The mean of the means of two injections of nine samples, the standard deviation of the nine means of two injections in parentheses.

d The mean of the means three injections of nine samples, the standard deviation of the means from three injections of nine samples in parentheses.

e Mean of the source means with the standard deviation of the mean in parentheses

F Gravimetric data described in Table 1.

g The mean of the means of three injections of five samples, the standard deviation of the means from three injections of five samples in parentheses.

**Table 4:** Certified concentrations for Aroclors in Methanol and Transformer Oil SRMs

SRM No.	Title	Mass fraction concentration^a^ mg kg^−1^	Volume fraction concentration^b^ mg L^−1^
3081	Aroclor 1016 in Methanol	17.13 ± 0.54	13.70 ± 0.44
3082	Aroclor 1232 in Methanol	5.25 ± 0.31	4.20 ± 0.25
3083	Aroclor 1242 in Methanol	16.36 ± 0.35	13.08 ± 0.29
3084	Aroclor 1248 in Methanol	6.89 ± 0.22	5.51 ± 0.18
3085	Aroclor 1254 in Methanol	7.08 ± 0.16	5.66 ± 0.13
3086	Aroclor 1260 in Methanol	6.18 ± 0.17	4.94 ± 0.14
3075	Aroclor 1016 in Transformer Oil	17.1 ± 1.0	15.2 ± 0.9
3076	Aroclor 1232 in Transformer Oil	4252 ± 114	3789 ± 106
3077	Aroclor 1242 in Transformer Oil	4102 ± 87	3656 ± 82
3078	Aroclor 1248 in Transformer Oil	3658 ± 161	3260 ± 146
3079	Aroclor 1254 in Transformer Oil	3579 ± 154	3190 ± 139
3080	Aroclor 1260 in Transformer Oil	1079 ± 98	962 ± 88

a Mass fraction data reported on the Certificate of Analysis; value and reported uncertainties are defined and discussed in text (see Sec. 3.3).

b Volume fraction data calculated by multiplying the certified mass fraction values by the measured densities of the methanol solution and transformer oil SRMs (see Sec. 3.3).

## Footnotes

1 Certain commercial equipment, instruments, or materials are identified in this paper to foster understanding. Such identification does not imply recommendation or endorsement by the National Institute of Standards and Technology, nor does it imply that the materials or equipment identified are necessarily the best available for the purpose.
